# Macon Medical Society

**Published:** 1885-02

**Authors:** 


### Society Reports.

### MACON MEDICAL SOCIETY.

Reported for THE ATLANTA MEDICAL AND SURGICAL JOURNAL.

STATED MEETING, December 2, 1884.

Dr. J. P. Stevens in the chair.

Dr. C. H. Hall read an essay on

MEMBRANOUS CROUP, of which the following is a synopsis :

Acute catarrhal laryngitis is an acute inflammation of mucous membrane, which only gives the products of a catarrhal inflammation, mucus, pus, and muco purulent fluid Acute croupous laryngitis is an acute inflammation which is ' accompanied by the exudation of an albuminous material upon mucous membrane of the larynx." Though one is a simple and easily managed disease, generally ending in rapid recovery, and the other the most malignant probably that we meet, yet in their incipiency it is frequently well nigh impossible to distinguish the one from the other. As a rule membranous croup develops slowly as compared with the ordinary catarrhal, and does not present the laryngial obstruction until the case is well advanced. The only certain diagnosis is the membrane, which can frequently be seen in the vomited matter, as also about the epiglottis. Laryngial diphtheria and membranous croup are by most American and a few European authors regarded as distinct diseases; most English, French and German contend for their identity, contending that exudation in the larynx is never the result of simple acute inflammation, but is always diphtheritic and constitutional. The advocates of duality of diphtheria and croupous laryngitis lay great stress on the exudation being on the mucous membrane in croup, and a part and parcel of it in diphtheria. Virchow, who originated this argument, now says that any distinction between the two membranes is not tenable; after abandoning his theory he promulgated the view that death of subjacent tissues was the characteristic and essential feature of diphtheria. "This distinction," says McKenzie, "was found to be no more satisfactory than the former, for cases came under observation which clinically answered to croup, but in which there was distinct death of tissues;" products of fibrinous degeneration. Rindfleish says, "pathological processes in both membranes are identical in the two diseases." Solis-Cohen, there are no anatomical differences. Wagner has declared that his preparations of croupous and diphtheritic membranes are very much alike. Steiner says, "according to E. Wagner, whose numerous and thorough investigations have shown that there is no sharp dividing line between diphtheria and croup—an opinion in which I most entirely agree." It has now been generally admitted that neither the eye nor the microscope can detect any difference between the two membranes. (McKenzie.) The weight of authority is in favor of the identity of the two diseases; this Bartholow admits, who is a believer in their duality. My own opinion is that they are identical. Prognosis unfavorable.

There is no specific treatment; general principles alone govern us. . The room of patients should be kept at a temperature of 70 to 75 degrees. Have steam constantly escaping into it by suitable apparatus, so as to keep the atmosphere moist; vaporize him freely and frequently; use oxygen if possible; inhalations of a solution of lactic acid; *unctus pro re nata*, recollecting their effect is only mechanical; whisky should be given liberally. The calomel treatment is, I think, pernicious, when large doses are given persistently. *Tracheotomy* may be resorted to if not postponed until the child is moribund.

Dr. Holt. I never saw any enlarged submaxillary glands in croup. They always occur in diphtheritic croup. I have seen recoveries in membranous, but never in diphtheritic croup. I have given calomel with no good effect. The best remedy in my experience has been turpentine in small and repeated doses; I do not know why. I used chloride of gold in one case, with no good effect. I believe that tracheotomy should be performed. I believe in the duality of the two diseases.

Dr. Stevens: In my opinion the weight of medical testimony goes to show a differentiation in the characteristics of membranous and diphtheritic croup. In the former we have a pure inflammation of the epithelial covering of the mucus membrane of the air passages, wherein an exfoliation of the false membrane is followed by another without any disintegration and destruction of the mucous tissue. In the second stage there is swelling and serious infiltration of the subepithelial textures of the larynx and vocal chords, inducing laryngeal stenosis, as well as a similar condition of the bronchial mucous membrane, not unfrequently resulting in the ejection of fibrinous casts of those tubes. The recovery of the patient from such attacks to a state of health is usually rapid, and uninterrupted by any dangerous sequelæ. The blood is not contaminated by any specific poison, and wonted vigor is soon re-established. In diphtheritic croup, Oertel clearly demonstrates that the inception of the membrane in the primary air passages is through the agency of *micrococci bacteria* germs that accomplish a lodgment there in the process of respiration, where a nidus is formed, and subsequently the blood becomes contaminated by the absorption of these microbites into the circulating current. Under the diphtheritic membrane the latter is loosened by a process of ulceration and destruction of tissue through the agency of bacteria, and reparation is made by a process of granulation. It is conceded, I believe, that these microbites are not found in the false membrane of membranous croup proper.

We cannot captiously ignore the depredations of these vegetable parasites. Koch has triumphantly vindicated the veritable existence of his comma bacillus, by experiments that show their invariable presence in the dejections of fatal cases of Asiatic cholera, and their invariable absence in those of cholera nostras. He has discomfited his traducers by exposing the inaccuracy of their microscopical investigations. Charles Bastian pertinaciously persisted for a long time in the truthfulness of his experiments in his advocacy of the spontaneous generation of living matter, until Tyndal annihilated his conclusions by his own unanswerable demonstrations of the fallacy of Bastian's experiments. The whole scientific world now admits that life is always antecedent to life. Admitting the microbitive origin of diphtheritic croup, the slow and tedious convalescence from its attack is easily accounted for, as well as the paralysis of the vocal chords, the pharynx, the ribs, limbs, sometimes of the heart, resulting in sudden death.

With reference to tracheotomy, statistics show that this operation is not as often performed as it should be. It is too long postponed. As soon as the third stage of the disease, that of asphyxia, is imminent, as manifested in the syanotic hue of the complexion and the general lethargy of the vital powers, indicating the overwhelming carbonization of the blood, the operation should be performed without delay. What the little sufferer needs above all things is more oxygen to neutralize the all-pervading carbonic acid, and give nature more time to rally her waning forces.

Steiner shows that out of 1,693 cases operated upon at the commencement of the stage of asphyxia nearly one-third recovered.

Dr. Williams: Owing to limited clinical experience on the subject I cannot express myself with entire conviction yet I am inclined to the opinion that the diseases are identical. That the diphtheritic membrane is formed *in* the mucosa, and cannot be removed without tearing the underlying tissues, leaving a bleeding surface, and that the croupous membrane is a coagulation *upon* the mucus membrane and can be scraped off without injury, was at first the opinion of Virchow and others. But since it has been demonstrated that the one gradually passes into the other, this can no longer be held as diagnostic.

Wagner, Rindfleisch and other pathologists contend that the microscopical appearances of the membranes are so nearly alike that it is difficult, if not impossible, to distinguish them. Dr. Cohen says, "We cannot establish any essential difference on anatomical grounds, the pathological products of the two affections being identical as far as our means of examining them are to be relied upon." The position of the affections, the one in the pharynx, the other in the larynx or trachea, cannot be, I think, diagnostic. Mackenzie says, "Croup is a disease which commonly commences in the pharynx and *only in about ten or twelve per cent.* of cases originates in the larynx or trachea." If we look at the anatomy of the pharynx, larynx and surrounding parts, we will see that the lymphatics of the tonsils, soft-palate and back of the pharynx communicate with the glands at the angle of the jaw, while those of the larynx and trachea only with the solitary glands at the great horn of the hyoid bone. Enlargement of the glands below the jaw cannot therefore be diagnostic of diphtheria; for same disease, which in the pharynx will cause enlargement of the glands at the angle of the jaw, will in the larynx enlarge the glands at the great horn of the hyoid.

In regard to albumenuria as diagnostic of diphtheria Professor DaCosta says: "On one symptom we cannot lay, as we now know, as much stress as might be supposed. The albumenuria, the recent elaborate report of the committee of the Medico-Chirurgical Society has taught us, is not always present in diphtheria owing to the early fatality of the malady. Again, in certain cases the mere dyspnœa of laryngitis may give rise to albumen in the urine." Paralysis is said to follow diphtheria and not croup. I have notes of a child in this city having sore throat with membranous exudation and some albumenuria, followed in four weeks by return of fluids through the nose and change in phonation. In the absence of a history of contagion or an epidemic I could not diagnose it diphtheria. McKenzie says, "Paralysis is rare in croup, because nearly all cases terminate fatally, but it is occasionally met with in those that survive"

As to Dr. Stevens's bacterian theory of disease, with all due respect to its advocates in high authority, I must say I have not yet given it my entire adherence. Bacteria can be and are found in the membranes of both affections, and it is yet to be decided whether there are special micrococci for diphtheria. We all know that organisms exist in all decay, animal or vegetable. No one denies that life is antecedent to life, nor do I "captiously ignore the depredations of vegetable parasites." Yet, until it can be undeniably demonstrated that bacteria are the sole cause of disease, I will wait, like doubting Thomas, to put my finger in the print of the nails left by these micrococci in diseased humanity.

Dr. McHatton: I am inclined to side with the dualists on the question of croup and diphtheria. In the former, the relation of the false membrane is only that of contact with the mucosa, there is no destruction of tissue, the capillaries do not rupture, it is not followed by cicatrices, there are no sequelæ, and finally it is not contagious. The latter is within as well as on the tissues, there is destruction of tissue with often sloughing, that involves even the muscles, followed by cicatrices and other sequelæ, it is contagious. There is no use going into the etiology of diphtheria, as to the question of the bacteria, that is yet unsettled. There are many drugs that dissolve the membrane of croup in theory but not in practice. Treatment should be symptomatic.

Tracheotomy is accepted by authorities, and should be performed as soon as the obstruction is sufficient to cause decided dyspnœa. I have seen two cases die, both of which, I think, might have been saved by the operation.

Dr. J. Emmett Blackshear: I do not feel that I can add anything of interest to what has been said, or throw any light whatever on this vexed question; but as it is perhaps desirable that every member present should express his opinion, I will simply say that my views are in harmony with those pathologists who regard the pathological process of the two diseases (diphtheria and croup) as identical, though the diseases may not be so, clinically. In croup, the deposit, as stated, is exterior to the body; in diphtheria it is imbedded in the tissues; death, in the former, is usually caused from stenosis of the larynx or trachea; in the latter from absorption of septic materials; but the want of identity in the deposit itself has never, to my mind, been satisfactorily proved.

As to treatment, I have nothing to recommend, as all treatment, in my hands, has proved futile. Tracheotomy, in my opinion, should always be resorted to when practicable.

Dr. McHatton reported the following case:

"I wish to report a case that is unique in my experience. I was called to see a girl of six, in perfect health, with the exception of an affection of the mouth, which I diagnosed as thrush, from the appearance, as well as the presence, of the oidium-albicans and an acid reaction in the secretions. Under borax, dissolved in glycerine, recovery was rapid. She had been sucking a bottle used by a baby affected with the disease, so it was probably directly contagious.

The peculiarity of the case is that it occurred in a perfectly healthy child. We know it is a disease usually developed during a depressed state of the vital forces, in which state it occurs at any age. It is at all times confined to squamous epithelium, consequently the opinion so prevalent that it occurs in the stomach and intestines is erroneous.

The oidium-albicans depends on an acid secretion for its full development, consequently the admixture of sugar in our medication is liable to offset the effect of the alkali.

